# *Helicobacter pylori* 23S rRNA gene A2142G, A2143G, T2182C, and C2195T mutations associated with clarithromycin resistance detected in Sudanese patients

**DOI:** 10.1186/s12866-021-02096-3

**Published:** 2021-02-03

**Authors:** Aalaa Mahgoub Albasha, Maram M. Elnosh, Esraa Hassan Osman, Duha M. Zeinalabdin, Amira A. M. Fadl, Musa Abdalla Ali, Hisham N. Altayb

**Affiliations:** 1 Department of Microbiology, College of Medical Laboratory Sciences, Sudan University for Science and Technology, Khartoum, Sudan; 2 Department of Medicine, The National Ribat University, Ribat University Hospital, Khartoum, Sudan; 3 Department of Microbiology, faculty of Medical Laboratory Science, University of Khartoum, Khartoum, Sudan; 4 Department of Biochemistry, Faculty of Sciences, King Abdulaziz University, Jeddah, 21452 Saudi Arabia

**Keywords:** *H. pylori*, Clarithromycin-resistance, A2142G, A2143G, Sudan

## Abstract

**Background:**

Clarithromycin resistant *Helicobacter pylori* (*H. pylori*) strains represent a worldwide health problem. These stains are usually carrying mutations within the 23S rRNA gene associated with clarithromycin resistance. This study aimed to detect *H. pylori* and clarithromycin resistant associated mutations from Sudanese patients with gastritis symptoms.

**Materials and methods:**

Two hundred and eighty-eight gastric biopsies were collected using gastrointestinal endoscopy from patients with gastritis symptoms in different hospitals in Khartoum state. *H. pylori* was detected by PCR using primer targeting 16S rRNA. Then allele-specific PCR and DNA sequencing were used to screen for the presence of A2142G and A2143G point mutations.

**Results:**

Out of 288 samples, *H. pylori* was detected in 88 (~ 30.6%) samples by 16 s RNA. Allele-specific PCR detected the variant A2142G in 9/53 (~ 17%) sample, while A2143G mutation was not found in any sample. The DNA sequencing revealed the presence of mutations associated with clarithromycin-resistance in 36% (9/25) of samples; the A2142G was present in one sample, A2143G in 5 samples and T2182C in 4 samples. Additionally, another point mutation (C2195T) was detected in 3 samples. There was no association of 23S rRNA gene point mutations with gender, age group, and patients’ geographical distribution.

**Conclusion:**

This study revealed a high frequency (36%) of mutations associated with clarithromycin resistance using DNA sequencing of the 23S rRNA gene’s V domain. This information should be taken into consideration to avoid eradication therapy failing.

## Introduction

*Helicobacter pylori* (*H. pylori*) is an exceptional bacterium in its ability to create permanent stomach colonization in untreated humans. Multiple factors contribute to the characteristic gut colonization, inflammation, alteration in the production of gastric acid, and tissue destruction caused by *H. pylori* [1]. The mechanisms by which *H. pylori* causes mucosal inflammation and damage are not well described but have both bacterial and host factors likely to be involved. Toxins and lipopolysaccharides produced by bacteria can damage the mucosal cells, and the ammonia released by the action of urease can also directly harm the cells [2]. In many developing countries, the infection rate has been reported to be as high as 70–80% [3]. *H. pylori* is responsible for more than 80% of peptic ulcer diseases, and 95% or more of duodenal ulcers [4]. Diagnostic testing is typically divided into invasive (endoscopic) and non-invasive approaches. The invasive diagnostic method involves endoscopic imaging, histology, rapid urease examination, culture, and molecular techniques. Non-invasive diagnostic tests include breathing tests for urea, antigen check for stools, and serological tests [5].

Elimination of *H. pylori* is based on successful treatment with a proton pump inhibitor (PPI), such as omeprazole, lansoprazole, and rabeprazole, and at least two antibiotics of clarithromycin (CLR), metronidazole (MTZ), amoxicillin (AMX), and tetracycline (TET). Combination therapy consisting of a PPI, CLR, and either AMX or MTZ for up to 14 days is one of the common approved first-line regimens [6]. As opposed to other macrolides, clarithromycin is used as an antibiotic against *H. pylori* due to its unusual acid stability. The antibiotic reversibly binds to domain II hairpin 35 and the domain V peptidyl transferase loop of the 23 s rRNA molecule inside the ribosomal subunit of the 50s. This binding prevents protein elongation by releasing peptidyl-tRNA prematurely from the acceptor site and thus effectively blocks the synthesis of bacterial proteins [7]. One mechanism by which *H. pylori* acquires antibiotic resistance is through vertical mutation transmission [8]. Clarithromycin-resistant strains also carry mutations within the gene 23S rRNA. Several studies have shown that A/G point mutations in Domain V at positions 2142 and 2143 or a T/C mutation in Domain VI of the 23S rRNA gene cause clarithromycin resistance [9–11]. The prevalence of antibiotic-resistant *H. pylori* is increasing worldwide [12]. Antibiotics resistance is the main factor of failure of *H. pylori* eradication therapies [13]. Clarithromycin resistance, in particular, has a major negative impact on the efficacy of the recommended first-line triple therapy of *H. pylori* [14]. In Sudan, there is very limited data on the prevalence of clarithromycin-resistant *H. pylori*. The aim of this study was to determine the *H. pylori* resistance to *clarithromycin* in Sudanese patients with gastritis symptoms.

## Materials and methods

### Collection of biopsy specimens

Gastric biopsies were collected from 288 patients, in which both the antrum and corpus had been sampled by endoscopy. Biopsies were collected by physicians from patients indicated for gastric endoscopy at different hospitals in Khartoum State (Omdurman Medical Military Hospital, Al-Amal National Hospital, Police Hospital, Ibn Sina Hospital, and Fedail hospital) at the period from June/2018 to January/2019.

### Preservation and processing of specimens

The specimens were immediately placed in thioglycollate broth, which provides anaerobic conditions until processing [15]. Manual grinding of biopsies took place using disposable material [16].

### Bacterial identification

The DNA of *Helicobacter pylori* has been extracted from specimens of the gastric biopsies using the guanidine chloride method [17]. Biopsies were ground by sterile blades and tips and then washed twice by phosphate buffer saline (PBS) to eliminate excess media. We add to the pellet 2 ml of lysis buffer, 10 μl of proteinase K, 1 ml of guanidine chloride, and 300 μl of ammonium (NH4) acetate, vortexed and incubated at 65 °C for 2 h. The mixture was cooled to ambient temperature, and then 2 ml of pre-cooled chloroform was applied, vortexed, and centrifuged for 5 min at 3000 rpm. The upper layer of the mixture was moved to a new tube, and 10 ml of absolute cold ethanol was applied, shaken, and held for 2 h or overnight at − 20 °C. The tube was then centrifuged for 15–20 min at 3000 rpm, the supernatant was carefully removed, and the tube was inverted for 5 min on a tissue paper. The pellet was washed with 70% ethanol 4 ml, centrifuged for 5 min at 3000 rpm. The supernatant was poured away, allowing the pellet to dry for 10 min. Then re-suspended into 50 μl of distilled water, briefly vortexed, and held overnight at − 20 °C. The extracted DNA was stored at − 70 °C until use.

### Polymerase chain reaction (PCR)

Two primer sets were used for the detection of the bacteria, targeting 16S rRNA (532 bp) [18],). Allele-specific PCR was used for the detection of A2142G and A2143G point mutations using four primers called FP-1, RP-1, RP2142G, and FP2143G (Table 1). When the strain is wild type (wt), neither RP2142G nor FP2143G anneals with the template and polymerase chain reaction (PCR) amplification proceeds between FP-1 and RP-1, resulting in a 320 bp amplicon. In the case of the presence of A2142G mutation, the PCR amplification primarily takes place between FP-1 and RP2142 G, which results in an amplicon of 238 bp. Similarly, in the case of the A2143G mutation, the PCR amplification goes between FP2143G and RP-1, resulting in an amplicon of 118 bp [19]. The primers were dissolved according to manufacturer guidelines to prepare 10 pmol/μl.

**Table 1:** Primers sequences and PCR protocols used in this study

Protocols	Primer name	Primer sequence (5′-3′)	Amplicon size (bp	References
1st	16 s RNA	GCTAA GAGA TCA GCC TAT GTCC TGGCAATCAGCGTCAGGTAATG	532	[18]
2nd	FP-1	TCGAAGGTTAAGAGGATGCGTCAGTC	320	[19]
	RP-1	GACTCCATAAGAGCCAAAGCCCTTAC		
	RP2142G	AGTAAAGGTCCACGGGGTATTCC	238	
	FP2143G	CCGCGGCAAGACAGAGA	118	

The first protocol used for amplification of 16S rRNA was as follows: initial activation at 94 °C for 3 min, followed by 35 cycles at 94 °C for 30s, 53 °C for 30s, and 72 °C for 45 s, and a final extension at 72 °C for 5 min (Table 1) [18].

The second protocol used for amplification of Allele-specific was as follows: initial denaturation at 95 °C for 5 min followed by 35 cycles of denaturation at 95 °C for 15 s, annealing at 60.5 °C for 20s, and extension at 68 °C for the 30s and a final extension of 2 min at 68 °C (Table 1) [19].

### DNA sequencing

A total of 25 PCR amplified products were sent for sequencing (by BGI, business, China) for both strands of PCR products. The pairwise alignment was done for successful sequences by BLAST, and then multiple sequence alignment was done by BioEdit software [20]. The sequences were compared with the 23S rRNA reference (U27270) and submitted to GenBank with accession numbers found in the additional files.

### Statistical analysis

The obtained data were analyzed using IBM SPSS statistics 20. The chi-square test was used to compare the correlations and associations between variables (*p*-value ≤0.05 considered significant).

## Results

### Demographic data

One hundred and twenty-eight (44.4%) were females, and one hundred sixty (55.6%) were males from two hundred and eighty-eight enrolled patients. They were divided into two age groups: adolescents (10) and adults (276). One hundred seventy-five (60.8%) specimens were collected from Khartoum city, and one hundred and thirteen (39.2%) specimens were collected from Omdurman city.

### Endoscopic findings

According to endoscopic findings by a physician, one hundred and ninety (66%) patients were diagnosed as gastritis, twenty-nine (10%) as a gastric ulcer (G. ulcer), twenty-five (9%) as a duodenal ulcer (D. ulcer), fifteen (5%) as esophagitis and twenty-nine (10%) were of normal finding.

### Detection of *H. pylori*

**Fig. 1:**
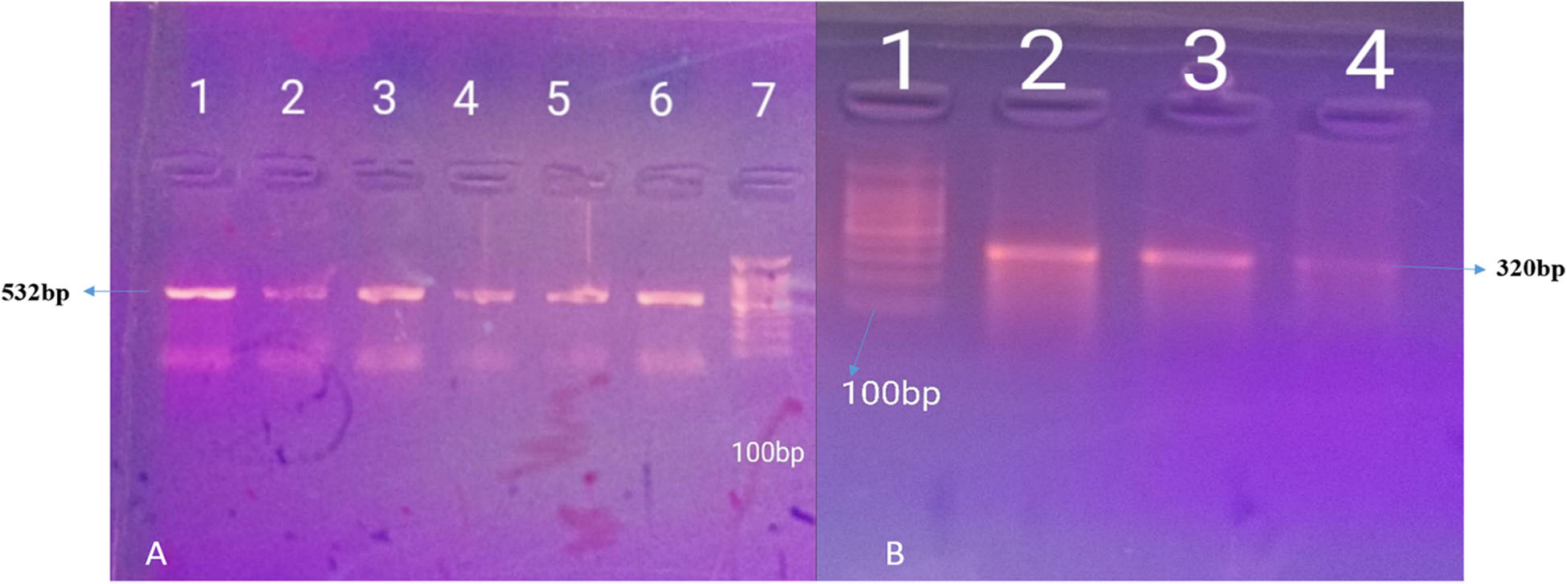
**a** PCR amplification of *H. pylori* detection genes 16S RNA in addition to wild type 23S rRNA on 1.5% agarose gel electrophoresis. A: Lane 7 marker (100-1500 bp), lanes 1 to 6 contain amplicons of16 s RNA (532 bp). **b**. Lane 1 marker (100-1500 bp). Lanes 2, 3, and 4 include amplicons of wt 23 s RNA (320 bp)

Out of 288 specimens investigated for the presence of *H. pylori* using primer targeting 16S rRNA by PCR, *H. pylori* were positive in 88/288 (~ 30.6%) specimens. Wild type (wt) 23S rRNA was detected in 62(21.5%) specimens, and both 16 s and wt 23 s RNA were positive together in 53(18.4%) specimens (Fig. 1).

### Association between the presence of *H. pylori* with the epidemiological and endoscopic findings

There was no significant correlation between the presence of *H. pylori*, epidemiological findings (Gender, age group, and geographical distribution of patients), and endoscopic findings in this study.

### Detection of A2142G and A2143G point mutations by allele-specific PCR

The A2142G point mutation was detected in 9/53 (~ 17%) specimens, whereas the second mutation (A2143G) was not detected in all samples.

### DNA sequencing results

**Fig. 2:**
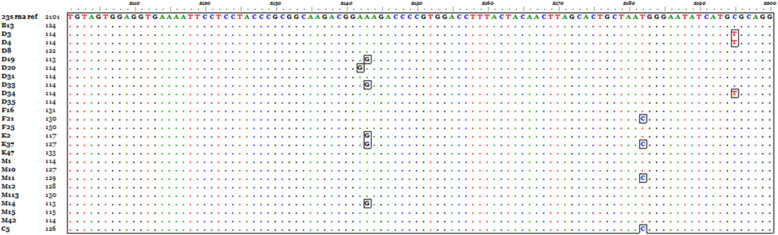
Multiple sequence alignment of 23S rRNA gene sequences compared to a reference gene, the mutant nucleotides appear in boxes

From twenty-five successfully sequenced samples, 12 samples exhibited different types of mutations at 23S rRNA gene, 9 (36%) samples showed mutations associated with clarithromycin resistance. And three samples reported with a mutation (C2195T) have no association with clarithromycin resistance. From the mutation associated with clarithromycin resistance, one sample showed the presence of A2142G point mutation, and the A2143G was found in 5 samples. Two other mutations (T2182C and C2195T) were detected in 4 and 3 samples, respectively. The A2142G was detected in one sample labeled D20, A2143G mutation detected in 5 samples D19, D33, K2, K37, and M14, T2182C mutation was detected in samples F11, K37, M11, and C5. The C2195T was detected in 3 samples D3, D4, and D34 (Fig. 2).

## Discussion

*H. pylori* infection is increasingly reported nowadays. Although patients are receiving treatment, the problem of antibiotic resistance still hinders their recovery. In particular, clarithromycin is the most prescribed antibiotic by physicians, and resistance to it may lead to treatment failure [21].

In this study, out of 288 patients with gastric pain, 66% were diagnosed as gastritis, 10% as gastric ulcer, 9% as duodenal ulcer, 5% as esophagitis, and 10% were normal patients. This finding agrees with other studies [22, 23], which found that gastritis is the most prevalent gastric disease.

Although culture isolation has been the standard method for the detection of the organism, but it may not be the most appropriate method for detection of *H. pylori* like organism due to cost, the special conditions required for specimen transport and growth, and the long interval between specimen harvest and test results, which delay treatment decision [15]. According to Malfertheiner [24] molecular technologies should be implemented as alternatives to traditional *H. pylori* Identification. In this study, the prevalence of *H. pylori* infection was 30.6% (88/288), using PCR targeting both 16S RNA genes. The latest prevalence rates of *H. pylori* among gastric biopsies from Sudanese patients were 21.1% using PCR targeting 16 S rRNA gene [25], and 22.2% using culture [26]. This variation could be attributed to that in our study, PCR was directly done from specimens without culturing step, which may minimise detection chance due to difficulties of cultivation.

*H. pylori* resist clarithromycin by specific mutations in the peptidyl transferase loop of the 23S rRNA molecule’s V domain [27–29]. Worldwide, the prevalence of clarithromycin-resistant strains of *H. pylori* is 19.4% [30]. Generally, countries with an antibiotic resistance rate of more than 20% alter their treatment strategies [31]. Our study revealed a higher frequency (36%) of mutations associated with clarithromycin resistance using DNA sequencing of V domain of 23S rRNA gene. While using the allele-specific PCR, the frequency of mutations associated with clarithromycin resistance in our specimens was 17% (9/53). These variations could be due to the low sensitivity of allele-specific PCR compared to DNA sequencing [32]. Also, in this study, allele-specific PCR targeted only two common mutations (A2142G and A2143G), while sequencing revealed all SNPs in the amplified region.

The point mutation A2142G was detected in 17% (9/53) of specimens using allele-specific PCR. This percentage is a noticeable amount compared with Tran [33] study in Vietnam, which found this mutation in about 3.6%, variation in the population may represent a critical factor.

Like Ghaith’s [23] study, point mutation A2143G was fallen to be detected by PCR although different PCR protocols were tried; this could be justified according to Cheng [34], which is that there is only one nucleotide difference between wild-type DNA and point mutation in DNA sequence. Therefore, the unusual mutations between large excess wild-type alleles are difficult to detect by traditional gene variation assays. In contrast, both mutations A2142G and A2143G appeared by DNA sequencing technique, and they are already known to cause reduced affinity of the ribosome for CLA [11].

As it appeared in our results, differences in detection methods has a larger impact. Fallen in the detection of A2143G mutiation by PCR and its appearance by DNA sequencing techniques may suggest that the percentage of clarithromycin resistance gene mutation may be more than the above results.

DNA sequencing also showed the presence of T2182C mutation in some specimens. According to Jung [35] suggestion, this mutation is nonspecific. In contrast, Khan [36] confirmed that this mutation is associated with clarithromycin. Besides, point mutation C2195T was detected by sequencing, and according to Fasciana [37], it has no relation with clarithromycin resistance.

## Data Availability

The datasets used and analyzed during the current study, in addition to accession numbers of GenBank submitted sequences are available at 10.6084/m9.figshare.13160144.v2

## Abbreviations

AMX: Amoxicillin
Bp: Base Pair
CLR: Clarithromycin
DNA: Deoxyribo-Nucleic Acid
MTZ: Metronidazole
PCR: Polymerase Chain Reaction
PPI: Proton Pump Inhibitor
rRNA: Ribosomal Ribonucleic acid
SPSS: Statistical package for social science
TET: Tetracycline
